# Does pulmonary rehabilitation address cardiovascular risk factors in patients with COPD?

**DOI:** 10.1186/1471-2466-11-20

**Published:** 2011-04-21

**Authors:** Nichola S Gale, James M Duckers, Stephanie Enright, John R Cockcroft, Dennis J Shale, Charlotte E Bolton

**Affiliations:** 1 Department of Physiotherapy, School of Healthcare Studies, Cardiff University, Heath Park, Cardiff, CF14 4XN, UK; 2 Section of Respiratory Medicine, School of Medicine, Wales Heart Research Institute, Cardiff University, Heath Park, Cardiff, CF14 4XN, UK; 3 Wales Heart Research Institute, School of Medicine, Cardiff University, Heath Park, Cardiff, CF14 4XN, UK; 4 NIHR Nottingham Respiratory Biomedical Research Unit, School of Clinical Sciences, University of Nottingham, Nottingham, NG5 1PB, UK

**Keywords:** COPD, pulmonary rehabilitation, arterial stiffness

## Abstract

**Background:**

Patients with COPD have an increased risk of cardiovascular disease. Whilst pulmonary rehabilitation has proven benefit for exercise tolerance and quality of life, any effect on cardiovascular risk has not been fully investigated. We hypothesised that pulmonary rehabilitation, through the exercise and nutritional intervention, would address these factors.

**Methods:**

Thirty-two stable patients with COPD commenced rehabilitation, and were compared with 20 age and gender matched controls at baseline assessment. In all subjects, aortic pulse wave velocity (PWV) an independent non-invasive predictor of cardiovascular risk, blood pressure (BP), interleukin-6 (IL-6) and fasting glucose and lipids were determined. These measures, and the incremental shuttle walk test (ISWT) were repeated in the patients who completed pulmonary rehabilitation.

**Results:**

On commencement of rehabilitation aortic PWV was increased in patients compared with controls (p < 0.05), despite mean BP, age and gender being similar. The IL-6 was also increased (p < 0.05). Twenty-two patients completed study assessments. In these subjects, rehabilitation reduced mean (SD) aortic PWV (9.8 (3.0) to 9.3 (2.7) m/s (p < 0.05)), and systolic and diastolic BP by 10 mmHg and 5 mmHg respectively (p < 0.01). Total cholesterol and ISWT also improved (p < 0.05). On linear regression analysis, the reduction in aortic PWV was attributed to reducing the BP.

**Conclusion:**

Cardiovascular risk factors including blood pressure and thereby aortic stiffness were improved following a course of standard multidisciplinary pulmonary rehabilitation in patients with COPD.

## Background

Chronic obstructive pulmonary disease (COPD) is an increasing cause of global morbidity and mortality [1] of which cardiovascular (CV) disease accounts for approximately 30% of the excess mortality in patients [2]. Such patients have multiple risk factors for CV disease including exposure to significant cigarette smoke, physical inactivity and metabolic disorders [3]. In addition, arterial stiffness measured by aortic pulse wave velocity (PWV) is an independent predictor of CV risk [4,5], which is increased in patients with COPD [6].

In the general population, addressing physical activity and nutritional optimisation, both components of multidisciplinary pulmonary rehabilitation, are associated with reducing the CV risks such as blood pressure (BP) and cholesterol [7]. Exercise, through biochemical, neural and hormonal alterations appear to improve vascular function and hence BP [8].

Demonstrable improvements in aortic PWV have been shown in non-COPD populations with short-term aerobic exercise training [9,10], however, the long-term benefits have not been established. In patients with COPD, a recent small study, demonstrated that four weeks of endurance exercise training reduced brachial artery PWV [11]. However, this peripheral measure has not been established as an independent CV prognostic factor [12], and the more robust aortic PWV which was not measured. Additionally, this was an isolated exercise intervention.

Multidisciplinary pulmonary rehabilitation is integral to the management of patients with COPD. Although the programme is short term, encompassing exercise, education and nutritional advice, its aim is to address lifestyle modification and several endeavours are currently addressing how benefits can be maintained. Demonstrable benefits in exercise capacity and quality of life are evident with improvements seen at 1 or 2 years following the course, even without a maintenance arm [13,14]. Whilst a range of overt co-morbidities are reported in patients entering rehabilitation, other than osteoporosis, they appear to exert little detriment to the standard outcome measures [15,16]. But how rehabilitation might address the CV co-morbidity has not been explored. We hypothesised that rehabilitation would improve the increased aortic PWV in patients with COPD (but with no overt CV disease or diabetes) together with blood pressure and other CV risk factors.

## Methods

### Study Design

The design was a prospective cohort study. Patients meeting the inclusion and exclusion criteria were recruited consecutively from the routine rehabilitation referral list at University Hospital Llandough. At baseline they were compared with control subjects who attended for one assessment only.

### Participants

Thirty-two patients with confirmed COPD, accepted for rehabilitation were recruited. Patients were clinically stable, defined as no change in dyspnoea, cough or sputum beyond day-to-day variability; or requirement for antibiotic or oral corticosteroid therapy in the preceding month. Subjects were excluded from the study (but not rehabilitation) if they had known ischaemic heart disease, cardiac failure, diabetes mellitus, malignancy, or any other inflammatory or metabolic condition, or were receiving oral corticosteroids, disease modifying or weight loss drugs. Twenty age- and sex-matched sedentary controls free from respiratory disease and other exclusions as above, but with a history of cigarette smoking were also recruited. Controls were spouses of patients or past volunteers who had agreed to consider future research. All subjects gave written, informed consent and the study had South East Wales Research Ethics Committee approval: 07/WSE04/05.

### Pulmonary Rehabilitation

The multidisciplinary pulmonary rehabilitation programme has been detailed previously [17]. Briefly, it is outpatient based, thrice weekly over seven weeks, with sessions lasting 2.25 hours. Each session comprises an educational element, aerobic and strength training, nutritional and behavioural therapy as well as relaxation technique and smoking cessation. A dietician provides individualised counselling and advice as well as group sessions to address weight loss, healthy diet or weight gain, as appropriate to the patient. Standard assessments include the St Georges respiratory questionnaire (SGRQ) [18], and the incremental shuttle walk test (ISWT), following a practice attempt. The ISWT was used to set the intensity of exercise training at 60-70% VO_2 _peak [19]. Patients who are on long-term oxygen therapy (LTOT) exercised with oxygen as per standard pulmonary rehabilitation conduct. Following rehabilitation, all patients are encouraged to continue exercise and opportunities exist at local leisure centres for patients to attend classes for respiratory patients.

### Study Procedures

#### Anthropometry and Lung Function

For all participants, height (m) and weight (kg) were measured barefoot, wearing lightweight clothing and body mass index (BMI) was calculated. All participants performed spirometry (Vitalograph, UK) to determine forced expiratory volume in 1 second (FEV_1_), forced vital capacity (FVC) and their ratio (FEV_1_/FVC) according to guidelines [20]. Patients were classified according to the Global Initiative for Chronic Obstructive Lung Disease (GOLD) criteria for severity of airway obstruction [1]. Resting oxygen saturations were recorded using a pulse oximeter (Pulsox-3iA, Konica-Minolta, Japan). All subjects self-completed a physical activity questionnaire [21].

### Haemodynamics

Subjects were assessed having fasted and abstained from caffeine, tobacco and inhaled short-acting β_2 _agonists for at least 6 hours. Peripheral systolic and diastolic BP was determined at the left brachial artery, and used to calculate mean arterial pressure (MAP) and pulse pressure (PP) (OMRON Corporation, Kyoto, Japan). Applanation tonometry using a high fidelity micromanometer (Millar Instruments Texas) was used to measure pulse waveforms at the radial, carotid and femoral sites, in relation to the R wave of a simultaneous ECG recording (SphygmoCor; AtCor Medical, Sydney, Australia). The radial pulse wave was used to generate a central arterial waveform, and augmentation index (AIx) was calculated as the difference between the first and reflected systolic peak as a percentage of pulse pressure, corrected to a heart rate of 75 beats per minute. Using integral software aortic (carotid - femoral) and brachial (carotid-radial) PWV was calculated as the speed of the pulse wave travelling between the 2 sites. Increased PWV and AIx indicate increased stiffness of blood vessels with aortic PWV being the preferable measure in this age group [5]. Prior to commencing pulmonary rehabilitation the reliability of the arterial stiffness measurements were tested in a subset of 19 patients. Assessments were repeated with a time interval of 1-3 weeks. Coefficients of reliability for aortic PWV were 0.90 and the intraclass correlation coefficients were 0.898. For brachial PWV, these values were 0.79 and 0.757 respectively and for AIx, 0.67 and 0.547. All assessments were completed by a single investigator, and waveforms were reviewed by an independent operator to verify quality and consistency.

### Biochemistry

A sample of venous blood was taken following the haemodynamic assessment, but prior to any exertion. Fasting glucose and cholesterol were determined within the Trust biochemistry laboratory according to standard procedure. Interleukin (IL)-6 was determined by ELISA (R&D Systems Europe, Abingdon, UK) with a minimum detectable limit of less than 0.70 pg/mL [22].

### Data Analysis

Data analysis was performed using the Statistical Package for the Social Sciences (SPSS, Chicago, IL), version 16.0. Skewed data were log_10 _transformed and checked for normality prior to analysis. Data are presented as mean and standard deviation (SD) unless stated. Patients and controls were compared using an unpaired *t *test and the chi squared test for categorical data. Relationships were investigated using Pearson's correlations.

Assessments pre and post rehabilitation were analysed using the paired *t *test for parametric data and linear regression was used evaluate the contribution of change in blood pressure to change in aortic PWV. The significance level for all statistical tests was set at p < 0.05. The sample size was based on previous published work from the department in patients with COPD [6]. For an 80% power, with a p < 0.05, 22 patients were required to detect a 15% reduction in aortic PWV from 11.4 m/s with a SD of 2.7 m/s.

## Results

Patients and controls were similar with respect to age, sex and BMI (Table 1). All participants had a history of cigarette smoking of at least 5 smoking pack-years. Five patients and two controls were current smokers and patients had a greater pack-year exposure. As expected, patients had impaired pulmonary function and lower resting oxygen saturations (p < 0.05). The mean PaO_2 _in patients at room air was 70 (10)mmHg, 4 patients were on LTOT and had been stabilised on this prior to commencement of rehabilitation. Thirteen patients classified as GOLD stage II and 19 patients were stage III/IV [1].

**Table 1:** Subjects characteristic in patients with COPD and control subjects

	Controls n = 20	Baseline Patients n = 32	p value
**Age (years) **#	64 (51-81)	65 (49-80)	0.730
**Gender (male%)**	7 (35%)	11 (34%)	0.964
**Height (m)**	1.67 (0.09)	1.62 (0.09)	0.083
**Weight (kg)**	74.1 (15.9)	67.0 (19.0)	0.166
**BMI (kg/m**^**2**^**)**	26.5 (3.9)	25.4 (6.4)	0.521
**Smoking pack-years #**	15 (5-50)	40 (5-90)	<0.001
**Current smokers**	2 (10%)	5 (15%)	0.595
**FEV**_**1 **_**(L)**	2.72 (0.66)	0.99 (0.39)	<0.001
**FEV**_**1 **_**(% predicted)**	108 (14)	45 (20)	<0.001
**FVC (L)**	3.50 (0.91)	2.18 (0.68)	<0.001
**FVC (% predicted)**	114 (13)	78 (22)	<0.001
**FEV**_**1**_**/FVC**	0.78 (0.06)	0.46 (0.15)	<0.001
**SpO**_**2 **_**(%) #**	97 (94-97)	96 (85-96)	<0.01
**Physical Activity Score (METs)#**	38 (28-78)	31 (25-58)	<0.001

Data presented as mean (SD), unless # = median (range).

*p < 0.05 and **p < 0.01 difference between baseline patients and control subjects

*Abbreviations*: **BMI **= body mass index; **FEV**_**1 **_= forced expiratory volume in 1 second; **FVC **= forced vital capacity; METs = metabolic equivalents; **SpO**_**2 **_= oxygen saturations pulse oximetry.

Of the 32 patients recruited at baseline, 31 (97%) of patients were taking a short-acting β_2_-agonist, 5 (16%) were taking a long-acting β-agonist alone, 25 (78%) were on combination inhaled therapy (long-acting β-agonist & corticosteroid), 30 (94%) were taking an anticholinergic bronchodilator.

Eight patients (25%) and four control subjects (20%) were taking antihypertensive medication, while five patients (16%) and six controls (30%) had been prescribed statins for hypercholesterolaemia. There were no other cardiac medication and there was no change to CV or respiratory maintenance medication was made during rehabilitation.

### At Commencement of Rehabilitation

#### Haemodynamic and Metabolic Data

Patients with COPD had greater aortic PWV than controls (p < 0.05) despite having similar MAP. There were 13 (41%) patients, 4 of whom were on prior treatment had a BP greater than the recommended, with either a systolic >140 or diastolic >90 mmHg. In parallel, 5 (25%) controls, 2 on treatment, also had elevated BP. Heart rate and IL-6 were also greater in patients than controls (Table 2). Although mean levels of glucose and lipids were similar between patients and controls, more patients had levels of total cholesterol above the optimal target for high risk individuals (>5.0 mmol/L), 22 (69%) compared with controls 10 (50%) [23].

**Table 2:** Baseline haemodynamic and metabolic data in patients with COPD and controls

	Controls n = 20	Patients n = 32	p value
**Aortic PWV (m/s)**	8.5 (1.4)	9.8 (2.7)	<0.05
**Brachial PWV (m/s)**	8.5 (1.2)	9.0 (1.7)	0.329
**AIx (%)**	26.9 (15.9)	32.3 (8.4)	0.156
**Peripheral SBP (mmHg)**	130 (16)	138 (19)	0.135
**Peripheral DBP (mmHg)**	79 (8)	82 (9)	0.247
**Peripheral PP (mmHg)**	51 (13)	56 (15)	0.196
**Peripheral MAP (mmHg)**	96 (10)	100 (11)	0.177
**Central SBP (mmHg)**	120 (16)	125 (18)	0.368
**Central DBP (mmHg)**	80 (8)	82 (11)	0.621
**Central PP (mmHg)**	40 (12)	47 (18)	0.133
**Central MAP (mmHg)**	97 (10)	104 (13)	0.055
**Heart rate (bpm)**	68 (11)	77 (12)	<0.01
**Fasting Glucose (mmol/L)**	5.2 (0.5)	5.0 (0.6)	0.236
**Total Fasting Cholesterol (mmol/L)**	5.2 (1.2)	5.6 (1.2)	0.360
**LDL (mmol/L)**	3.0 (1.2)	3.3 (1.1)	0.322
**HDL (mmol/L)**	1.6 (0.7)	1.7 (0.5)	0.504
**Cholesterol/HDL ratio**	3.8 (1.5)	3.5 (1.0)	0.373
**Triglyceride (mmol/L)**	1.2 (0.6)	1.1 (0.4)	0.591
**IL-6 † (pg/ml)**	1.3 (3.1)	3.8 (2.1)	<0.001

Data presented as mean (SD), unless otherwise stated, **† **= Geometric mean.

*Abbreviations*: **AIx **= augmentation index; **DBP**= diastolic blood pressure; **HDL **= high-density lipoprotein; **IL-6 **= interleukin 6; **LDL **= low-density lipoprotein; **MAP **= mean arterial pressure; **PP **= pulse pressure; **PWV **= pulse wave velocity; **SBP **= systolic blood pressure.

### Anthropometry

Although mean BMI was similar in patients with COPD and control subjects, BMI was more heterogeneous in the patients. Six patients (no controls) were underweight (BMI <20 kg/m^2^), and 8 patients (3 controls) were obese (BMI ≥30 kg/m^2^) [24].

In all subjects and in patients alone, as expected, aortic PWV was related to age (r = 0.579 and r = 0.593 respectively, both p < 0.001); as well as aortic PWV related to central and peripheral systolic BP and peripheral MAP (all p < 0.05). Aortic PWV did not relate to other clinical variables; FEV_1_, oxygen saturations or smoking pack-years. Although patients reported less physical activity in the preceding month than controls this was not related to aortic PWV.

### The Effect of Pulmonary Rehabilitation

Of the 32 patients, 22 completed rehabilitation - for this group of completers, median (range) number of attendances was 18 (14-20) out of 20. There was no difference in age, gender, baseline lung function, haemodynamic variables or body composition between patients who completed study assessments and those who did not. Reasons for non-completion were illness, personal constraints or exclusion from the study due to commencement of oral corticosteroids. One of the five patients who smoked at baseline stopped smoking during rehabilitation.

**Figure 1:**
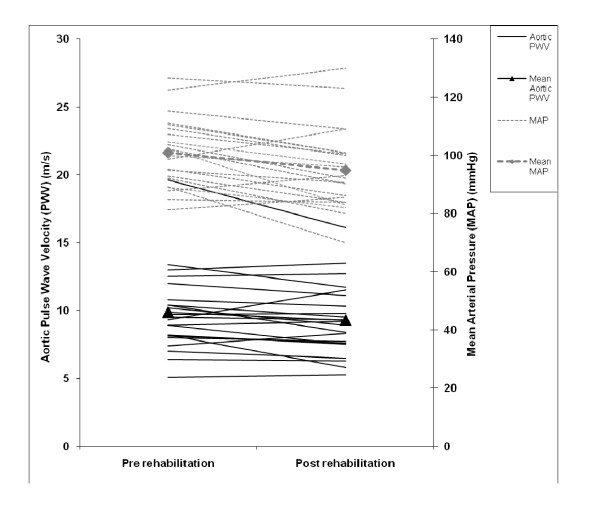
**Change in Aortic Pulse Wave Velocity and Mean Arterial Pressure with Pulmonary Rehabilitation in patients with COPD**. Solid lines represent the change in aortic pulse wave velocity (PWV), triangles denote the mean change in aortic PWV. Dotted lines show the change in mean arterial pressure (MAP), diamonds indicate mean change in MAP.

Following rehabilitation aortic PWV reduced from 9.8 (3.0) to 9.3 (2.7) m/s (p < 0.05) (Figure 1) and peripheral BP decreased markedly (all p < 0.05) (Table 3). Using linear regression, following adjustment for the change in central MAP the improvement in aortic PWV was attenuated. The reduction in BP occurred in both patients who had an elevated BP (11/22 had elevated BP) and those who commenced with a BP within the recommended range. Following rehabilitation, 3 of the 11 patients with high BP at baseline no longer met the criteria for hypertension at completion despite no change in pharmacological therapy. There was a modest reduction in total cholesterol (5.6 (1.2) to 5.4 (1.1) mmol/L p < 0.05), and 2 of the 15 patients no longer met the criteria for high cholesterol after rehabilitation. Brachial PWV and AIx were unchanged with rehabilitation.

**Table 3:** Effect of pulmonary rehabilitation in patients with COPD

	Start of rehabilitation	End of rehabilitation	p value
**Aortic PWV (m/s)**	9.8 (3.0)	9.3 (2.7)	<0.05
**Brachial PWV (m/s)**	9.0 (1.7)	8.9 (1.6)	0.535
**AIx (%)**	33.9 (5.3)	33.6 (8.8)	0.833
**Peripheral SBP (mmHg)**	138 (20)	128 (24)	<0.01
**Peripheral DBP (mmHg)**	83 (9)	78 (12)	<0.01
**Peripheral PP (mmHg)**	55 (15)	49 (19)	<0.05
**Peripheral MAP (mmHg)**	101 (12)	95 (14)	<0.001
**Central SBP (mmHg)**	126 (21)	120 (23)	<0.05
**Central DBP (mmHg)**	83 (12)	79 (12)	<0.05
**Central PP (mmHg)**	43 (14)	41 (18)	0.471
**Central MAP (mmHg)**	103 (14)	95 (14)	<0.001
**Heart Rate (bpm)**	76 (12)	76 (14)	0.933
**Fasting Glucose (mmol/L)**	5.0 (0.7)	4.8 (0.8)	0.079
**Total Fasting Cholesterol (mmol/L)**	5.6 (1.2)	5.4 (1.1)	<0.05
**LDL (mmol/L)**	3.4 (1.1)	3.3 (1.2)	0.389
**HDL (mmol/L)**	1.7 (0.5)	1.6 (0.4)	0.050
**ISWT (m)**	190 (70)	274 (76)	<0.001
**SGRQ Total Score**	56.5 (13.9)	44.9 (16.1)	<0.001

Data presented as mean (SD)

*Abbreviations*: **AIx **= augmentation index; **DBP**= diastolic blood pressure; **HDL **= high-density lipoprotein; **IL-6 **= interleukin 6; **ISWT = **Incremental shuttle walk test; **LDL **= low-density lipoprotein; **MAP **= mean arterial pressure; **PP **= pulse pressure; **PWV **= pulse wave velocity; **SBP **= systolic blood pressure; **SGRQ **= St Georges respiratory questionnaire.

Overall there was no change in glucose, IL-6 or BMI. However as expected, traditional rehabilitation outcome measures - ISWT and SGRQ improved, although these did not relate to change in aortic PWV. There were 19/22 patients who exceeded the minimum clinically significant improvement of 47.5 m [25] and 17 patients exceeded the minimum clinically significant difference of 4 units for total SGRQ score [18].

## Discussion

This is the first study to evaluate the effect of a standardised multidisciplinary pulmonary rehabilitation programme on central arterial haemodynamic risk factors in patients with COPD. Pulmonary rehabilitation improved several cardiovascular risk factors including aortic PWV, an independent predictor of CV risk in the general population, blood pressure and cholesterol [4,5]. A recent study of four weeks of endurance cycling, in ten patients with COPD, demonstrated improved peripheral arterial stiffness measured by brachial PWV in improved secondary to systolic BP improvement [11]. In comparison, the seven untrained patients had no significant haemodynamic change. Our findings corroborate this study using the 'gold standard' aortic PWV as the primary variable, with exercise being part of a pulmonary rehabilitation programme, which is already integrated in COPD management. Similar to Vivodtzev et al, we demonstrated a reduction in systolic BP with the intervention and the fact that systolic BP explained the reduction in aortic PWV does not detract from the actual improvement of aortic stiffness, whatever the mechanism with pulmonary rehabilitation. There was no change in brachial PWV or AIx found which may be a consequence of the different properties of central and peripheral arteries, nor were either markedly increased compared to controls in the present study and both are inferior measurements to aortic PWV in this age group of subjects [5,12].

The clinical relevance of the change in aortic PWV in this study is uncertain as a clinically significant reduction has yet to be fully established. Certainly, an increase in aortic PWV of 1 m/s corresponds to an adjusted risk increase of 15% in CV mortality [26], but the reverse is less clear. A 10 mmHg reduction in systolic BP is likely to improve CV risk - a recent meta-analysis of pharmacological intervention, showed using a meta-regression analysis, that a 5 mmHg reduction in systolic BP equated to a 13% reduction in risk of CV death, myocardial infarction or stroke [27].

Although limited data exists in subjects with COPD, the haemodynamic benefits of aerobic exercise have been demonstrated in non-respiratory conditions. In subjects with hypertension 4 weeks of exercise resulted in a 9% reduction in aortic PWV [28]. This is somewhat higher than the present study but may be explained by a younger population, without respiratory impairment. The antihypertensive effects of aerobic exercise have been well established in both normotensive and hypertensive individuals using standard brachial sphygomanometry [29]. Additionally, cardiac rehabilitation, which provides exercise and lifestyle modification to cardiac patients has been shown to reduce BP and cholesterol [30]. All of these studies support the short-term reduction in CV risk factors demonstrated with pulmonary rehabilitation in this present study of patients with COPD.

The mechanism of improved haemodynamics following exercise has not been established, but may include increased shear stress with improved structural compliance, nitric oxide related vasodilation and reduced sympathetic drive [31]. The latter potential mechanism was reported in patients with COPD after six weeks of aerobic exercise training [32], but the effects on BP were not recorded. In non-COPD individuals with hypertension, exercise training decreased BP along with reduced vascular resistance and sympathetic nervous activity [29].

We and others have previously reported increased arterial stiffness in patients with COPD compared to controls [6,33,34]. It was important however, to confirm if this was seen in the selected patients referred to rehabilitation compared to previous reported groups of COPD patients [6,28,29]. Why patients with COPD have increased aortic PWV has not been fully established, however they have multiple risk factors for CV disease including a history of cigarette smoking, physical inactivity, nutritional and metabolic abnormalities as well as airflow obstruction [35]. As in the general population we report aortic PWV was related to age and BP in both patients and in the whole subject group. However, aortic PWV was greater in patients compared to age matched controls in the setting of a similar MAP, hence suggesting that there are other contributing factors.

Aortic PWV has previously been associated with impaired lung function in both COPD subjects [6] and a general population of men [36]. Pulmonary function has also been associated with risk factors for metabolic disease [37,38], which appears to be at increased prevalence in patients with COPD [39]. An increased systemic inflammatory state is characteristic in COPD, and has been related to increased CV risk in the general population. In this study, the inflammatory mediator IL-6 was increased in patients with COPD, but it was not attenuated by rehabilitation, concurring with previous reports of rehabilitation intervention [40].

The limitations of this study include the small sample size and a high rate of drop out from the study over the course of pulmonary rehabilitation. Whilst we still met our sample size, the mean aortic PWV at baseline was lower than previously reported and which was used in our power calculation [6], perhaps reflecting the characteristics of a subgroup of patients with COPD who are deemed suitable for pulmonary rehabilitation, but additionally a larger proportion were female. This lower baseline value is likely to, at least partially, account for why we did not see the reported 15% change in aortic PWV with rehabilitation. The study could be criticised for the inclusion of patients and controls with hypertension however, where feasible, we tried to mimic patients entering the standard pulmonary rehabilitation programme whilst excluding clear confounders such as prior ischaemic heart disease or diabetes. It is plausible that adherence to the pulmonary rehabilitation programme could have improved adherence to medication in general given pulmonary rehabilitation focuses on lifestyle factors. No control COPD group was included as it would not have been ethical to deny patients with COPD access to pulmonary rehabilitation. Considering conducting a "comparable" course for the controls encompassing the multidisciplinary aspects of rehabilitation, whilst ideal, was open to several other confounders, not least trying to equate the exercise across courses. It was not possible to match smoking exposure between patients and controls. However, Maclay and colleagues has already reported the difference in aortic PWV between matched smokers with and without COPD [34]. The change in haemodynamics appeared across the range at baseline, pointing away from regression to the mean. Having presented these findings, assessment of haemodynamics before and after pulmonary rehabilitation on a larger scale is required.

## Conclusion

This study confirms the presence of multiple cardiovascular risk factors in patients with COPD undergoing multi-disciplinary pulmonary rehabilitation, and is the first to show that a standardised programme appears to improve the cardiovascular risk profile. Pulmonary rehabilitation may be an opportunity to identify and treat cardiovascular and metabolic dysfunction and therefore may provide additional benefit lead to patients. Further research is required to corroborate and determine the longer term implications of these haemodynamic changes. The clinical impact of the findings reported is potentially transferable across rehabilitation programmes and reinforces the known functional and health status improvements that pulmonary rehabilitation already delivers.

## Abbreviations

AIx: augmentation index; BMI: body mass index; BP: blood pressure; COPD: chronic obstructive pulmonary disease; CV: cardiovascular; DBP: diastolic blood pressure; FEV_1_: forced expiratory volume in 1s; FVC: forced vital capacity; GOLD: Global Initiative for Chronic Obstructive Lung Disease; HDL: high density lipoprotein; ISWT: incremental shuttle walk test; IL-6: interleukin; LDL: low density lipoprotein; LTOT: long-term oxygen therapy; MAP: mean arterial pressure; PaO_2_: arterial partial pressure of oxygen; PP: pulse pressure; PWV: pulse wave velocity; SBP: systolic blood pressure; SGRQ: St Georges respiratory questionnaire; SD: standard deviation; SpO_2_: peripheral oxygen saturation; VO_2 _peak: peak oxygen consumption.

## Competing interests

The authors declare that they have no competing interests.

## Authors' contributions

NG helped design the study, conducted the clinical assessments, analysed and drafted the manuscript; JD contributed to the clinical evaluation of subjects, interpretation and writing; SE contributed to the interpretation and writing and provided academic support to NG; JC helped design the study, interpret and contributed to the writing; DS helped design the study, assisted with interpretation and contributed to the writing; CB helped design the study, assisted with conduct of study, analysis and interpretation and helped write the manuscript. All authors read and approved of the final manuscript.

## Pre-publication history

The pre-publication history for this paper can be accessed here:

http://www.biomedcentral.com/1471-2466/11/20/prepub
